# 12-Item Pruritus Severity Scale: Development and Validation of New Itch Severity Questionnaire

**DOI:** 10.1155/2017/3896423

**Published:** 2017-10-02

**Authors:** Adam Reich, Agnieszka Bożek, Katarzyna Janiszewska, Jacek C. Szepietowski

**Affiliations:** ^1^Department of Dermatology, Venereology and Allergology, Wroclaw Medical University, Wroclaw, Poland; ^2^Department of Dermatology, University of Rzeszów, Rzeszów, Poland

## Abstract

**Introduction:**

A validated assessment of pruritus intensity is an important but still difficult clinical problem due to a subjective nature of this sensation.

**Objective:**

The aim of this study was the creation and validation of new itch severity questionnaire assessing pruritus intensity.

**Material and Methods:**

A total of 148 patients with pruritic dermatoses were asked to assess pruritus intensity using 12-Item Pruritus Severity Score (12-PSS) and Visual Analogue Scale (VAS). Patients were also asked to complete the Dermatology Life Quality Index (DLQI) and Hospitality Anxiety and Depression Scale (HADS). Test-retest comparison of 12-PSS was conducted in 102 subjects who completed the itch questionnaire twice with the 3- to 5-day interval.

**Results:**

We have created the 12-PSS assessing pruritus intensity (two questions), pruritus extent (one question) and duration (one question), influence of pruritus on concentration and patient psyche (four questions), and scratching as a response to pruritus stimuli (four questions). A maximum scoring was 22 points. The results showed strong consistency (Cronbach *α* coefficient 0.81). A significant correlation was observed with VAS (*r* = 0.58, *p* < 0.001) and quality of life level according to DLQI (*r* = 0.53, *p* < 0.001). Test-retest comparison in 102 subjects revealed a satisfactory reproducibility of achieved results (ICC = 0,72).

**Conclusions:**

The newly developed pruritus severity questionnaire may be used in daily clinical practice in the future.

## 1. Introduction

Pruritus is defined as an unpleasant sensation leading to the desire to scratch. It can be distinguished as acute (<6 weeks) or chronic (i.e., pruritus lasting 6 or more weeks). Chronic pruritus, which can be distressing and often refractory to treatment, is associated with many diseases. It is a primary symptom of various dermatological diseases, including atopic dermatitis, psoriasis, and urticaria. It is also a common feature of several systemic diseases, such as chronic kidney failure, cholestatic liver diseases, human immunodeficiency virus (HIV) infection, and haematopoietic disorders [1–3]. Chronic pruritus is often accompanied by a high level of psychiatric comorbidities and sleep disturbances with considerable impact on the health-related quality of life (QoL) [4]. Pruritus is a subjective symptom and, therefore, it is difficult to be accurately measured in an objective way. However, developing and validating measures are becoming increasingly important in dermatological research. The development of new therapeutic approaches requires an objective assessment of diseases. The assessment of the antipruritic effect is, to date, based solely on patient reports on the course of pruritus or measurements of scratch movements [4]. Assessing the intensity of pruritus as objectively as possible is extremely important, not only for research purposes, but also in clinical practice. However, an ideal scoring system for all the purposes is not available. Currently, several assessment methods are available to evaluate pruritus severity: monodimensional Pruritus Severity Scales (e.g., Visual Analogue Scale (VAS), Numerical Rating Scale (NRS), and Verbal Rating Scale (VRS) [5]), multidimensional questionnaires (like the 5-D Pruritus Scale [2], Pruritus Severity Scale [3], and the Eppendorf Pruritus Questionnaire [6]), and the measurement of sensory threshold or scratching activity [7].

Based on the recently published consensus, it is recommended to use the VAS or NRS in daily clinical practice and at least two independent methods in research studies or clinical trials [4]. However, no widely accepted, standardized, and validated questionnaire for objective measuring of pruritus is currently available [8]. The Visual Analogue Scale (VAS) is considered as the most reliable and valid pruritus assessment scale. However, it is clear that the use of a single measure of pruritus intensity does not ensure an adequate and comprehensive assessment of chronic pruritus, as pruritus may differ not only regarding its intensity, but also, for example, its duration or extent [8]. Numerous different methods were used for assessment of the intensity of pruritus in the past. However, most of them did not undergo proper validation and reliability testing. The lack of reliable pruritus assessment tools that would evaluate various aspects of pruritus has motivated us to develop and validate a new pruritus severity questionnaire that would assess pruritus intensity, scratching response, and the influence of pruritus on patient's mood and concentration.

## 2. Materials and Methods

### 2.1. Subjects

A total of 148 patients (81 females and 67 males) between ages 18 and 91 years (mean age, 50.0 ± 15.7 years) with chronic dermatological pruritus (>6 weeks) were included in the study. Pruritus was associated with lichen planus in 78 cases (52.7%), psoriasis in 31 cases (20.9%), atopic dermatitis in 25 cases (16.9%), and other skin diseases in 14 cases (9.5%). All patients were Caucasians. The patients were recruited from inpatients and outpatients attending regular visits at the Department of Dermatology, Venereology and Allergology, Wroclaw Medical University. Inclusion criteria for the study included (1) pruritus associated with one of the chronic skin diseases, (2) chronic pruritus defined as lasting for at least 6 weeks, (3) age over 18 years, (4) ability to give informed consent, and (5) ability to complete the questionnaire. All included subjects agreed to participate in the study and signed a written informed consent prior to any further procedures.

### 2.2. Development of 12-Item Pruritus Severity Scale

Creation of the 12-Item Pruritus Severity Scale (12-PSS) was based on the review of the literature concerning existing pruritus assessment tools, discussion with patients, and our own clinical experience in the diagnosis and treatment of patients with chronic pruritus. The development of our questionnaire has also been based on a consensus paper of the Special Interest Group (SIG) initiated by members of the International Forum on the Study of Pruritus (IFSI) to determine which domains and structure of pruritus questionnaires need to be implemented to assess chronic pruritus in a better way [8]. The 12-PSS is a one-page instrument consisting of 12 items that assess different aspects of pruritus. The items were grouped into five domains: pruritus intensity (2 questions: Q9, Q10), pruritus extent (1 question: Q11), frequency and duration of pruritus (1 question: Q1), impact of pruritus on daily activities and mood (4 questions: Q2–Q5), and scratching assessment as a response to pruritus (4 questions: Q6–Q8 and Q12) (Table 1). Total scores can range from 3 (minimal pruritus) to 22 (most severe pruritus).

### 2.3. Data Collection

After providing written informed consent, patients were asked about their demographics, medical diagnosis, course of disease, and comorbidities. Afterwards, all subjects meeting inclusion criteria completed a questionnaire package which included the 12-PSS, the VAS [9], and the Dermatology Life Quality Index (DLQI) [10]. In addition, the Hospitality Anxiety and Depression Scale (HADS) were given to 75 patients to assess the level of anxiety and depressive mood [11]. Only patients who did not start any treatment for current exacerbation of the skin disease were included into the study. All assessments in the first round were completed prior to treatment initiation. One hundred and two patients who returned 3–5 days after enrollment for reassessment were asked to complete the questionnaires to evaluate test-retest reliability.

### 2.4. Statistical Analysis

Statistical analysis was performed using Statistica 12.0 (Statsoft, Kraków, Poland). Groups were compared with Student's* t*-test or with analysis of variance (ANOVA) with Scheffé post hoc test, where appropriate. Correlations between the individual components of 12-PSS and the total score of 12-PSS were calculated using Spearman's rank order correlation test. Spearman's correlation coefficient (*ρ*) was interpreted as follows: *ρ* = 0–0.1, no correlation; *ρ* = 0.1–0.29, weak correlation; *ρ* = 0.3–0.49, moderate correlation; *ρ* = 0.5–0.7, strong correlation; *ρ* > 0.7, very strong correlation [10]. The correlations between the 12-PSS, VAS, and DLQI were calculated using Pearson's correlation coefficient and interpreted as follows: *r* ≤ 0.3, weak correlation; *r* > 0.3 but *r* < 0.5, moderate correlation; and *r* ≥ 0.5, strong correlation [12]. Differences between the first and the second assessments were verified with paired Student's* t*-test. Intraclass correlation coefficient (ICC) was calculated to assess test-retest reliability. ICC < 0.4 indicated poor reliability, 0.4 ≤ ICC < 0.75 fair to high reliability, and ICC ≥ 0.75 excellent reliability [13]. Internal consistency was determined using Cronbach's alpha coefficient. Coefficient scores >0.7 generally indicate high internal reliability [14]. *p* values less than 0.05 were considered significant.

## 3. Results

### 3.1. Distribution and Discriminant Validity of 12-Item Pruritus Severity Scale

**Figure 1:**
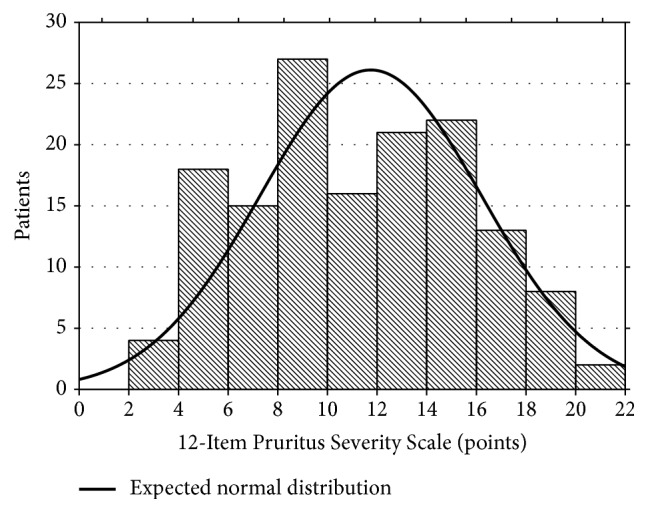
Distribution of the total scorings of 12-Item Pruritus Severity Score.

The mean scoring of 12-PSS for all patients was 11.7 ± 4.5 points (range 3–21 points). The total scoring showed wide, almost equal distribution of achieved responses, except the most outer results (Figure 1). Neither ceiling nor bottom effect was observed.

**Figure 2:**
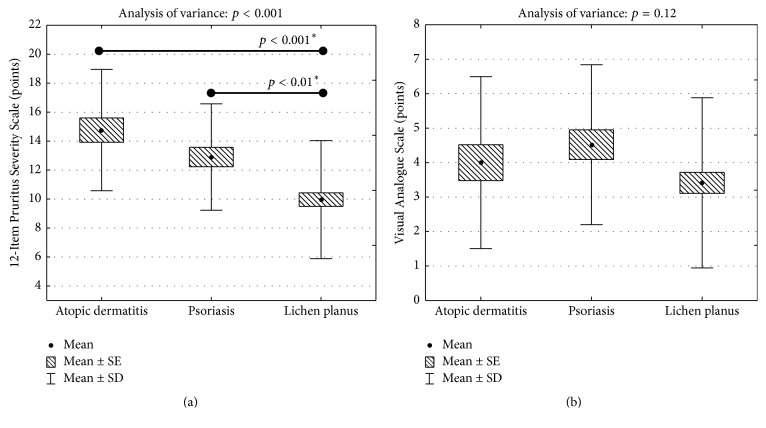
(a) Comparison of pruritus severity assessed according to 12-PSS in various dermatoses (SE: standard error of mean; SD: standard deviation, ^*∗*^*p* values according to Scheffé post hoc test). (b) Comparison of pruritus severity assessed according to VAS in various dermatoses (SE: standard error of mean; SD: standard deviation).

**Figure 3:**
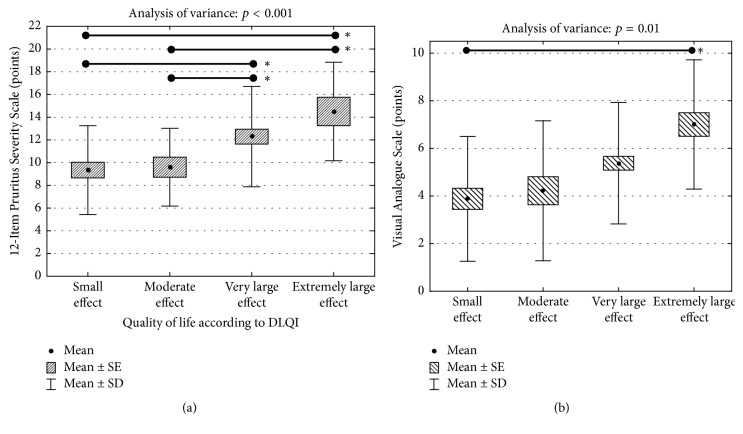
Comparison of pruritus severity assessed according to 12-PSS in patients with various impairments of health-related quality of life (DLQI: Dermatology Life Quality Index, SE: standard error of mean, SD: standard deviation, ^*∗*^*p* < 0.01 acc. to Scheffé post hoc test). (b) Comparison of pruritus severity assessed according to VAS in patients with various impairments of health-related quality of life (DLQI: Dermatology Life Quality Index, SE: standard error of mean, SD: standard deviation, ^*∗*^*p* = 0.05 acc. to Scheffé post hoc test).

Comparison of the mean pruritus severity between various dermatoses revealed that with 12-PSS we were able to detect significant differences between subjects with atopic dermatitis (mean pruritus severity: 14.8 ± 4.2 points), psoriasis (12.9 ± 3.7 points), and lichen planus (10.0 ± 4.1 points; *p* < 0.001) (Figure 2(a)). Interestingly, such difference was not observed, when pruritus severity was measured with VAS (atopic dermatitis: 4.0 ± 2.5 points, psoriasis 4.5 ± 2.3 points, and lichen planus 3.4 ± 2.5 points; *p* = 0.12) (Figure 2(b)). With 12-PSS, we were also able to detect significant differences between patients having various levels of QoL impairment assessed according to DLQI (*p* < 0.001) (Figure 3(a)). Based on these findings, we may conclude that 12-PSS is able to detect changes between patients suffering from pruritus of different intensity supporting the discriminant validity of 12-PSS. The VAS scoring was much less discriminative regarding various QoL categories (Figure 3(b)).

### 3.2. Internal Consistency

The assessment of internal consistency was performed based on 148 questionnaires. The results of our study have shown that the different items of the questionnaire are interrelated with one another. Cronbach's alpha coefficient was 0.81 indicating strong internal consistency.

### 3.3. Convergent Validity

Correlation of individual components and the total score of the 12-PSS by Spearman's correlation coefficient showed statistically significant values. Most of the questions showed strong correlation with the total score (*r* > 0.5). Only one question (Q7) demonstrated weak, albeit statistically significant correlation (*r* < 0.29) and two questions (Q4 and Q6) moderate correlation (Table 2). Overall, it could be concluded that the 12-PSS shows satisfactory convergent validity.

### 3.4. Correlations of the 12-Item Pruritus Severity Scale with the VAS, DLQI, and HADS

**Figure 4:**
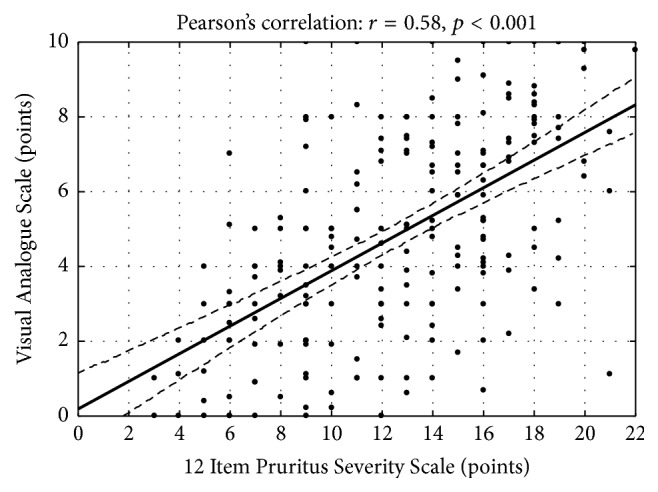
Correlation between the 12-PSS and the VAS scoring.

**Figure 5:**
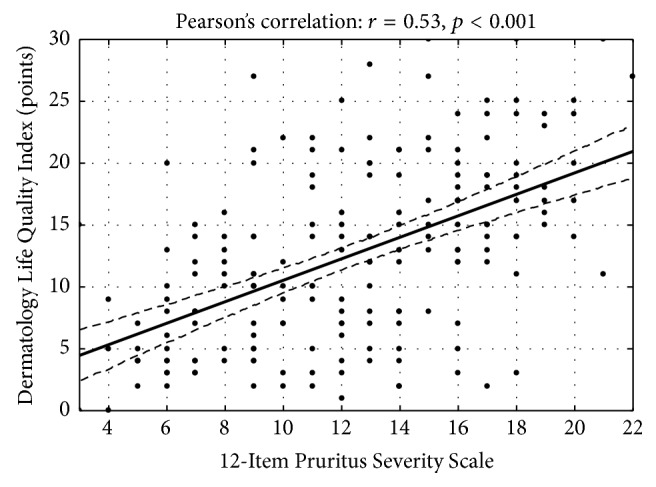
Correlation between the 12-PSS and the DLQI scoring.

The 12-PSS demonstrated significant correlation with the VAS, DLQI, and anxiety and depression level assessed with HADS. The correlation between the 12-PSS and the VAS was strong (*r* = 0.58, *p* < 0.001) (Figure 4). The 12-PSS also showed significant correlation between the impairment of QoL (*r* = 0.53, *p* < 0.001) (Figure 5) and HADS scoring (for anxiety: *r* = 0.37, *p* = 0.001; for depression: *r* = 0.28, *p* = 0.01). These latter correlations could be assessed as weak to moderate, suggesting that 12-PSS reflected influence of pruritus on patients but provided slightly different information than the scales used as comparators. Interestingly, the VAS, which solely assesses pruritus intensity, showed only moderate correlation with DLQI (*r* = 0.46, *p* < 0.001) and no significant correlation with HADS components (anxiety domain: *r* = 0.16, *p* = 0.16; depression domain: *r* = 0.16, *p* = 0.17). Based on these observations, it could be concluded that the 12-PSS is able to catch a more complex influence of pruritus on patient well-being than the VAS. In addition, 12-PSS also correlated with pruritus duration (*r* = 0.39, *p* < 0.001). The mean 12-PSS scoring for patients with pruritus lasting <1 year was 10.3 ± 4.2 points and for those with pruritus lasting ≥1 year 12.6 ± 4.4 points (*p* = 0.001).

### 3.5. Test-Retest Comparison

The reproducibility of the 12-PSS in the group of 102 subjects was high. The intraclass correlation coefficient (ICC) between the 12-PSS scores obtained in two assessments (I and II) was 0.72. Significant differences were observed only in three questions, where the second assessment demonstrated significantly lower values which might represent some variations in pruritus intensity (Table 3). However, we also cannot exclude the possibility that some patients applied treatments during this time leading to some improvement of skin condition and lowering of pruritus intensity.

## 4. Discussion

The new pruritus severity questionnaire (12-PSS) has been developed as a simple, multidimensional method for assessing pruritus intensity. The evaluation of the psychometric properties of the questionnaire has revealed that the measure demonstrates high internal consistency and convergent validity, a significant correlation with the VAS and quality of life level, and a high reproducibility. This indicates that the questionnaire is a reliable and valid measure of pruritus severity.

The test-retest reliability of the pruritus severity questionnaire was high. However, over a 3–5-day interval, the scores of some questions demonstrated lower values in the second assessment. This can be explained by spontaneous pruritus intensity fluctuations within few days. However, this interval is necessary for the patient not to remember the previous answers. It is also possible that within this period of time patients might apply some treatment modalities, even unintentionally, that decreased pruritus intensity, which might influence the achieved results. Taking this into consideration, it could be assumed that the questionnaire has a satisfactory sensitivity to change, but this requires further research.

The 12-PSS is easy to understand and to complete for subjects and is rather short and quick to be completed (usually less than 3 minutes). The questionnaire contains questions regarding different aspects of pruritus. It delivers data about localization, duration, frequency and intensity of pruritus, scratch response, disability, and quality of life impairment in patients with pruritus. It provides important information about relevant characteristics of pruritus and discriminates between different types of pruritus. It may be able to detect changes in pruritus over time. According to the Special Interest Group, our pruritus questionnaire considers the patient's perspective, the physician's perspective, and needs of various measurements in clinical trials [8].

The VAS is the most reliable scale to quantify pruritus currently. However, relying on a single measure is not sufficient for evaluation of pruritus. The VAS is adequate in assessing the intensity of symptoms but does not provide information about other aspects of pruritus, like the impact of pruritus on the QoL. However, it is of importance as chronic pruritus can significantly reduce patients QoL. Therefore, additional instruments assessing the psychiatric comorbidities, impact on patient's QoL and patient satisfaction should also be integrated into the routine care of chronic pruritus. For this reason, there are a variety of tools available that assess the QoL of affected patients. The DLQI is a widely used instrument which has already been validated [15]. However, the use of additional scale assessing the impact of pruritus on patient's QoL is time-consuming and sometimes impossible to implement in everyday practice. The data from our study has demonstrated that our pruritus severity questionnaire significantly correlates with the DLQI. Furthermore, it contains questions regarding the influence of pruritus on patient's daily life, mood, and concentration. The developed pruritus severity questionnaire can be successfully used as an appropriate tool to evaluate the severity of pruritus and the impact of pruritus on the QoL at the same time.

In conclusion, the developed pruritus severity questionnaire is a reliable measure that has been validated in patients with chronic pruritus. It may be a useful tool both in clinical trials and routine daily practice to qualify patients for the antipruritic treatment and to assess the efficacy of therapy.

## Figures and Tables

**Table 1:** 12-Item Pruritus Severity Scale.

	Question	Possible answers	Scoring
(1)	How often did you feel pruritus within the last 3 days?	(i) All time	3 points
		(ii) All morning/afternoon/evening/night long itch episodes	2 points
		(iii) Occasionally, short itch episodes	1 point

(2)	Did pruritus hinder your ability to do simply things, like watching TV, hearing music, etc.?	(i) Yes (ii) No	1 point 0 points

(3)	Did you feel irritated or nervous because of your itching?	(i) Yes (ii) No	1 point 0 points

(4)	Did your pruritus cause you depressed?	(i) Yes (ii) No	1 point 0 points

(5)	Did your pruritus impede your work or learning abilities?	(i) Yes (ii) No	1 point 0 points

(6)	Did you scratch your skin because of itching?	(i) Yes (ii) No	1 point 0 points

(7)	Did scratching bring you relief?	(i) Yes (ii) No	0 points 1 point

(8)	Were you able to refrain from scratching?	(i) Yes (ii) No	0 points 1 point

(9)	Did you wake up during last night because of pruritus?	(i) No (ii) Yes, 1-2 times (iii) Yes, 3-4 times (iv) Yes, 5 and more times	0 points 1 point 2 points 3 points

(10)	Could you assess the severity of your pruritus within last 3 days?	(i) Very mild (ii) Mild (iii) Moderate (iv) Severe (v) Very severe	1 point 2 points 3 points 4 points 5 points

(11)	Could you indicate pruritus location?	(i) Single locations of pruritus (ii) Large body areas (iii) Generalized pruritus	1 point 2 points 3 points

(12)	Are excoriations or other scratch lesions present?	(i) Yes (ii) No	1 point 0 points

**Table 2:** Spearman's rank correlation coefficients (*ρ*) for the each item (Q) score and the total score of the 12-PSS.

	*N*	*ρ*	*p*
Q1 and total score	148	0.76	<0.0001
Q2 and total score	148	0.62	<0.0001
Q3 and total score	148	0.62	<0.0001
Q4 and total score	148	0.49	<0.0001
Q5 and total score	148	0.68	<0.0001
Q6 and total score	148	0.32	<0.0001
Q7 and total score	148	0.2	0.01
Q8 and total score	148	0.53	<0.0001
Q9 and total score	148	0.72	<0.0001
Q10 and total score	148	0.8	<0.0001
Q11 and total score	148	0.66	<0.0001
Q12 and total score	148	0.5	<0.0001

**Table 3:** Reproducibility of results achieved with pruritus severity questionnaire in 102 patients (based on paired Student's *t*-test, results demonstrated as means ± standard deviations).

	First assessment	Second assessment	*p*
Question 1	2.0 ± 0.7	1.8 ± 0.8	0.02
Question 2	0.5 ± 0.5	0.5 ± 0.5	0.66
Question 3	0.8 ± 0.4	0.8 ± 0.4	0.57
Question 4	0.6 ± 0.5	0.6 ± 0.5	1.0
Question 5	0.5 ± 0.5	0.6 ± 0.5	0.32
Question 6	0.9 ± 0.2	0.9 ± 0.3	0.32
Question 7	0.3 ± 0.5	0.3 ± 0.5	0.57
Question 8	0.7 ± 0.5	0.6 ± 0.5	0.42
Question 9	1.3 ± 1.1	1.0 ± 0.9	<0.05
Question 10	3.4 ± 1.1	2.9 ± 1.2	0.01
Question 11	2.1 ± 0.7	2.0 ± 0.8	0.17
Question 12	0.7 ± 0.5	0.6 ± 0.5	0.32
Total score	13.8 ± 4.1	11.3 ± 4.5	<0.001
